# Supplementary material for: The Small RNA Universe of Capitella teleta
Source: Front Mol Biosci. 2022 Feb 25;9:802814. doi: 10.3389/fmolb.2022.802814 (PMC8915122; doi:10.3389/fmolb.2022.802814)
Supplement: Supplementary file 1 [file DataSheet1.ZIP › Supplement/known/CAPTEscaffold_114_8284.pdf]

Provisional ID	:	CAPTEscaffold_114_8284
Score total	:	4495.7
Score for star read(s)	:	3.9
Score for read counts	:	4486.1
Score for mfe	:	1.1
Score for randfold	:	1.6
Score for cons. seed	:	3
Total read count	:	8811
Mature read count	:	8182
Loop read count	:	34
Star read count	:	595

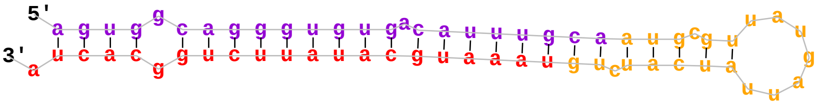

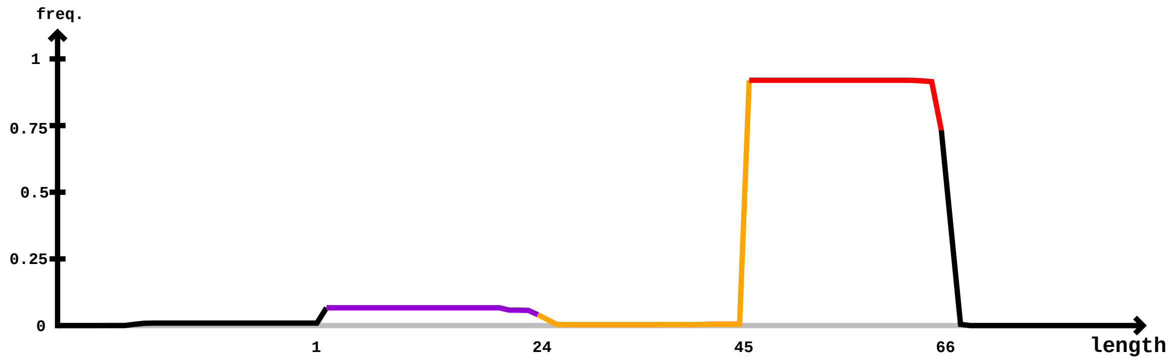

Star **Mature**

```
5'- guuaugauuccgcugaucacugggucaguggcagggugugacauuugcaaugcguuaugauuaucaucuguaaaugcauauucuggcacuaaucuggucgaccucucacua  -3'   obs
    guuaugauuccgcugaucacuggguca guggcagggugugacauuugcaaugcguuaugauuaucaucuguaaaugcauauucuggcacuaaucuggucgaccucucacua         exp
    guuaugauuccgcugaucacugggucaguggcagggugugacauuugcaaugcguuaugauuaucaucuguaaaugcauauucuggcacuaaucuggucgaccucucacua         known
    ...............((((((((.((((.(((((((((.((((((((((.((.......))))).)))))))))))))))))).)))).))))))).))..........   reads   mm   sample
    ...augauuccgcugaucacuggguc.....................................................................................   1       0    seq
    ....ugauuccgcugaucacuggguc.....................................................................................   1       0    seq
    ......auucUgcugaucacuggguc.....................................................................................   1       1    seq
    ......auuccgcugaucacuggguc.....................................................................................   39      0    seq
    .......Guccgcugaucacuggguc.....................................................................................   1       1    seq
    .......uuccgcugaucacuggguc.....................................................................................   33      0    seq
    ........uccgcugaucacuggguc.....................................................................................   7       0    seq
    ..........................aguggcagggugugacauu..................................................................   77      0    seq
    ..........................aguggcaggAugugacauu..................................................................   1       1    seq
    ..........................aguggcagggugugacauuu.................................................................   3       0    seq
    ..........................aguggcagggugugacauuug................................................................   7       0    seq
    ..........................aguggcagggugugacauuugc...............................................................   140     0    seq
    ..........................Gguggcagggugugacauuugc...............................................................   2       1    seq
    ..........................aguggcagggugugGcauuugc...............................................................   1       1    seq
    ..........................agGggcagggugugacauuugc...............................................................   1       1    seq
    ..........................agAggcagggugugacauuugca..............................................................   1       1    seq
    ..........................aguggcagggugugaGauuugca..............................................................   1       1    seq
    ..........................Gguggcagggugugacauuugca..............................................................   2       1    seq
    ..........................aguggcagggugugacauuugca..............................................................   355     0    seq
    ..........................aguggcagggugugacauuugcaU.............................................................   1       1    seq
    ..........................aguggcagggugugacauuugcaUu............................................................   2       1    seq
    ..........................aguggcagggugugacauuugcaau............................................................   1       0    seq
    .............................................Agcaaugcguuaugauuau...............................................   1       1    seq
    .................................................augcguuaugauuaucaucu..........................................   6       0    seq
    .................................................augcguuaugauuaucaucug.........................................   27      0    seq
    .................................................augcguuaugauuaucaucugu........................................   1       0    seq
    .............................................................uaucaucuguaaaugcauauucuggcacuaa...................   1       0    seq
    .................................................................aucuguaaaugcauauucuggc........................   2       0    seq
    ..................................................................ucuguaaaugcauauucuggc........................   2       0    seq
    ..................................................................ucuguaaaugcauauucuggcU.......................   3       1    seq
    ..................................................................ucuguaaaugcauauucuggca.......................   11      0    seq
    ..................................................................ucuguaaaugcauauucuggcacu.....................   1       0    seq
```

Star   Mature

```
guuaugauuccgcugaucacugggucaguggcagggugugacauuugcaaugcguuaugauuaucaucuguaaaugcauauucuggcacuaaucuggucgaccucucacua
....................................................................uguaaaugcauauucuggcacu.....................    1      0      seq
......................................................................uaaaugcauauucuggca.......................    3      0      seq
......................................................................uaaaugcauauucuggcac......................    27     0      seq
......................................................................uaaaugcauauucuggcGcu.....................    1      1      seq
......................................................................uaaaugUauauucuggcacu.....................    25     1      seq
......................................................................Aaaaugcauauucuggcacu.....................    9      1      seq
......................................................................uaaaugcauauucuggcaAu.....................    1      1      seq
......................................................................uaaaugcauauucCggcacu.....................    1      1      seq
......................................................................uaaauCcauauucuggcacu.....................    1      1      seq
......................................................................uaaaugcauauucuggcacG.....................    2      1      seq
......................................................................uaaaugcaAauucuggcacu.....................    1      1      seq
......................................................................uaaaugcUuauucuggcacu.....................    1      1      seq
......................................................................uaUaugcauauucuggcacu.....................    1      1      seq
......................................................................uaaaugcauauAcuggcacu.....................    2      1      seq
......................................................................uaaaugcauauuGuggcacu.....................    2      1      seq
......................................................................uaaaugcauauucAggcacu.....................    2      1      seq
......................................................................uaaaugcauauucuggcacu.....................    1564   0      seq
......................................................................Caaaugcauauucuggcacu.....................    1      1      seq
......................................................................uaaaAgcauauucuggcacu.....................    2      1      seq
......................................................................uaaaugcauauuAuggcacu.....................    1      1      seq
......................................................................uaaaCgcauauucuggcacu.....................    1      1      seq
......................................................................uaaaugcauaAucuggcacu.....................    1      1      seq
......................................................................uaaaugcauauuGuggcacua....................    1      1      seq
......................................................................uaaaugcauauucuAgcacua....................    2      1      seq
......................................................................uaaaugcauauGcuggcacua....................    1      1      seq
......................................................................Aaaaugcauauucuggcacua....................    29     1      seq
......................................................................uaaaugcauauucuggcacGa....................    1      1      seq
......................................................................uaaUugcauauucuggcacua....................    1      1      seq
......................................................................uaaaugcauauucuggcacuU....................    5      1      seq
......................................................................uaaaugcauauuAuggcacua....................    2      1      seq
......................................................................Gaaaugcauauucuggcacua....................    1      1      seq
......................................................................uaaauCcauauucuggcacua....................    1      1      seq
......................................................................uaaaugcauaAucuggcacua....................    1      1      seq
......................................................................uUaaugcauauucuggcacua....................    1      1      seq
......................................................................uaaaugcauauucuggcUcua....................    2      1      seq
......................................................................uaaaugUauauucuggcacua....................    2      1      seq
......................................................................uaaaugcauauuUuggcacua....................    1      1      seq
......................................................................uaaaugAauauucuggcacua....................    10     1      seq
......................................................................uaaaugcauauucAggcacua....................    1      1      seq
......................................................................uaaaAgcauauucuggcacua....................    5      1      seq
......................................................................uaaaugcaAauucuggcacua....................    3      1      seq
......................................................................uaGaugcauauucuggcacua....................    1      1      seq
......................................................................uaaaugcauauucuggUacua....................    1      1      seq
......................................................................uaaaugcUuauucuggcacua....................    2      1      seq
......................................................................uaaaugcauauucuggcacuG....................    9      1      seq
......................................................................uaaaGgcauauucuggcacua....................    1      1      seq
......................................................................uaaaugcauauucuggcacua....................    4696   0      seq
......................................................................uaaaugcauGuucuggcacua....................    1      1      seq
......................................................................uaaaugcauauucuggcGcua....................    1      1      seq
......................................................................uaaaugcauauucCggcacua....................    2      1      seq
......................................................................uaaaugcauauCcuggcacua....................    1      1      seq
......................................................................uaaaugcauauAcuggcacuaa...................    1      1      seq
......................................................................uaaaugcauauucuggcacuaC...................    8      1      seq
......................................................................uaaaugcaCauucuggcacuaa...................    1      1      seq
......................................................................uaaaugcauaCucuggcacuaa...................    1      1      seq
......................................................................uaaaugAauauucuggcacuaa...................    5      1      seq
......................................................................uaaaugcauUuucuggcacuaa...................    1      1      seq
......................................................................uaaaugcauauuAuggcacuaa...................    2      1      seq
......................................................................Aaaaugcauauucuggcacuaa...................    8      1      seq
......................................................................Caaaugcauauucuggcacuaa...................    1      1      seq
......................................................................uaaaugcauauucuggcacuaU...................    27     1      seq
......................................................................uaaaugUauauucuggcacuaa...................    2      1      seq
......................................................................uaaaugcauauucuggcGcuaa...................    1      1      seq
......................................................................uaaaAgcauauucuggcacuaa...................    2      1      seq
......................................................................uGaaugcauauucuggcacuaa...................    2      1      seq
......................................................................uaaaugcauauucuggcacuaa...................    1639   0      seq
......................................................................uaaaugcUuauucuggcacuaa...................    1      1      seq
......................................................................uaaaugcauauucuggcacuGa...................    1      1      seq
......................................................................uaaaugcauauucuggcacuaaA..................    28     1      seq
......................................................................uaaaugcauauucuggcacuaaG..................    1      1      seq
```

```
                          Star                                      Mature
guuaugauuccgcugaucacugggucaguggcagggugugacauuugcaaugcguuaugauuaucaucuguaaaugcauauucuggcacuaaucuggucgaccucucacua
......................................................................uaaaugcauauucuggcacuaau..................   12   0   seq
.......................................................................Uaaugcauauucuggcacua....................   2    1   seq
.......................................................................Uaaugcauauucuggcacuaa...................   1    1   seq
```
